# Chemiosmotic energy for primitive cellular life: Proton gradients are generated across lipid membranes by redox reactions coupled to meteoritic quinones

**DOI:** 10.1038/s41598-019-48328-5

**Published:** 2019-08-28

**Authors:** Daniel Milshteyn, George Cooper, David Deamer

**Affiliations:** 1 Department of Biomolecular Engineering, University of California, Santa Cruz, CA 95064 USA; 2 NASA Ames Research Center, Moffett Field, CA 94035 USA

**Keywords:** Biophysics, Chemistry

## Abstract

Transmembrane proton gradients coupled to, and maintained by, electron transport are ubiquitous sources of chemiosmotic energy in all life today, but how this system first emerged is uncertain. Here we report a model liposome system in which internal ferricyanide serves as an oxidant and external ascorbate or dithionite provide a source of electrons to electron carriers embedded in liposome membranes. Quinones linked the donor to the acceptor in a coupled redox reaction that released protons into the vesicle internal volume as electrons were transported across the membranes, thereby producing substantial pH gradients. Using this system, we found that one or more quinones in extracts from carbonaceous meteorites could serve as coupling agents and that substantial pH gradients developed in the acidic interior of liposomes. If amphiphilic compounds present on the prebiotic Earth assembled into membranous compartments that separate reduced solutes in the external medium from an encapsulated acceptor, quinones can mediate electron and proton transport across the membranes, thereby providing a source of chemiosmotic energy for primitive metabolic reactions.

## Introduction

**Figure 1:**
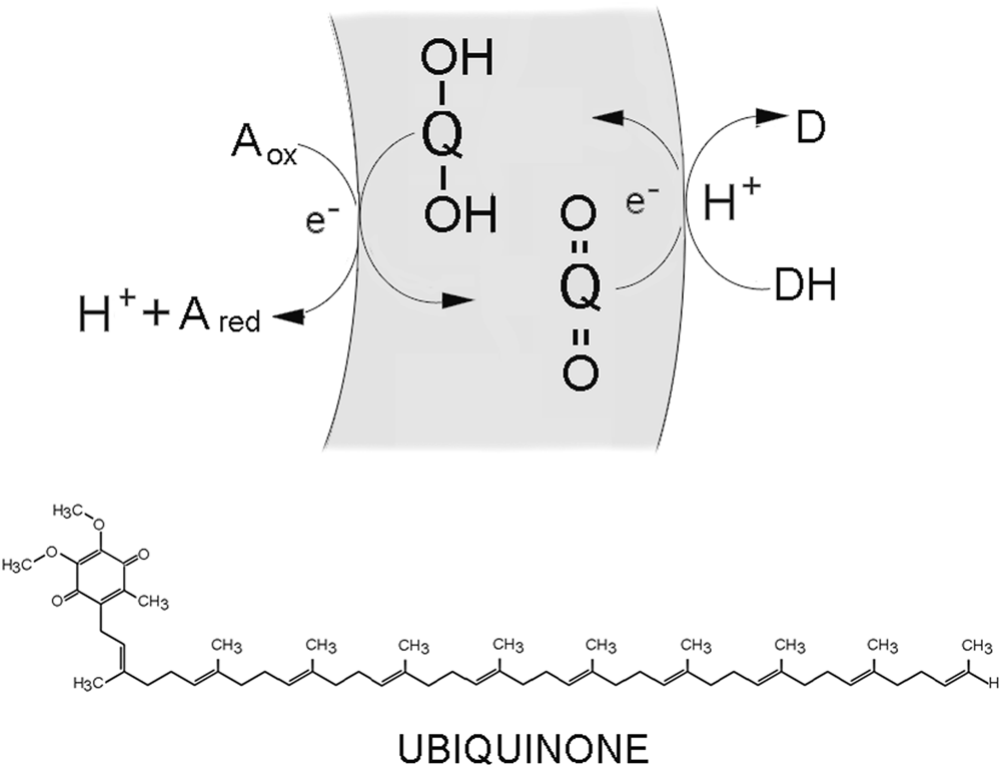
Role of quinones in coupling electron transport to proton transport across bilayer membranes. An external donor source of reducing power (DH) transfers both electrons and protons to a quinone (Q) which diffuses to the other side of the membrane. The electrons reduce an oxidant (A_ox_) such as an iron compound that does not accept protons in the reaction, and the protons are then released into the interior of the compartment. The structure of ubiquinone which functions in mitochondria as an electron transport cofactor is shown.

All forms of life require an energy source linked to metabolic reactions to sustain cellular processes such as metabolism, growth and reproduction. In life today, the energy source involves coupling of electron transport to the production and maintenance of an electrochemical proton gradient^1^. In order to understand how these bioenergetic systems originated, we can ask what compounds were likely to have been present to participate in prebiotic chemistry and serve as components in primitive electron transport processes. Enzymes play the most prominent role in biological electron transport, but are too complex to be candidates for prebiotic ingredients on the early Earth. On the other hand, quinones have been conserved in their roles as electron carrier cofactors in most biological electron transfer pathways, and were likely delivered to the prebiotic Earth from meteoritic infall in the form of polycyclic aromatic hydrocarbon (PAH) derivatives. Bioenergetic electron transfer involving various quinones is ubiquitous in all three domains of life, including ubiquinone in mitochondria, plastoquinone in chloroplasts, and menaquinone in bacteria^2^. In these electron transport systems, the quinones act as an electron carrier when they diffuse across membranes after being reduced by an external electron donor and then oxidized by an encapsulated electron acceptor. In this cycle (Fig. 1) quinones act as lipid soluble hydrogen shuttles because they partition into the non-polar hydrocarbons of the lipids and diffuse within the bilayers between external and internal volumes as they pick up and drop off electrons and protons.

Quinones such as phenanthrenedione and anthracenedione are PAH derivatives that have been detected in carbonaceous meteorites^3,4^. It has also been demonstrated that quinone compounds can be synthesized in simulated interstellar ice cloud environments through photochemical reactions between PAH compounds and UV radiation^5–8^. A quinone can also be synthesized from anthracene by a clay-catalyzed reaction^9,10^. Delivery of these compounds by the infall of interplanetary dust and meteorite impacts may have provided quinones for the first functional living systems. In addition, nitrogen-containing heteorocyclic compounds are known meteorite constituents^3,4,8^. Although phenazine, the di-nitrogen heterocycle described in this work, has not been searched for in meteorites, it is a plausible prebiotic compound given the suite of similar ancient compounds.

Earlier studies showed that phenazine methosulfate (PMS) can act as an electron carrier to mediate electron transfer between ascorbate, an electron donor, and ferricyanide, an electron acceptor, across a phospholipid bilayer to produce significant pH gradients^11^. The magnitude of the pH gradients was monitored by 9-aminoacridine (9AA), a fluorescent probe. The goal of the research described here was to use this system to determine whether compounds in meteorites can serve as electron carriers that allow proton gradients to develop in lipid vesicles. The meteorites used in this study, Murchison, Murray, Mighei and ALH 85013, all belong to the CM (“M” for Mighei) group of carbonaceous meteorites (“chondrites”). They are also petrologic type 2, which means that they experienced significant aqueous alteration on their parent asteroid bodies. These meteorites were chosen, in part, because CM2 meteorites generally possess significant amounts of organic material, some of which are soluble in aqueous and organic solvents, while other kerogen-like compounds are insoluble. Murchison, in particular, is the most extensively studied CM since falling in 1969: it possesses a significant complement of identified compounds including monocarboxylic acids composed of at least eleven carbons^8^ that are capable of self-assembly into membranous compartments. For updated classification, descriptions, and background information on all meteorites, see the Meteoritical Society Bulletin database^12^.

## Results

### Phenazine methosulfate

**Figure 2:**
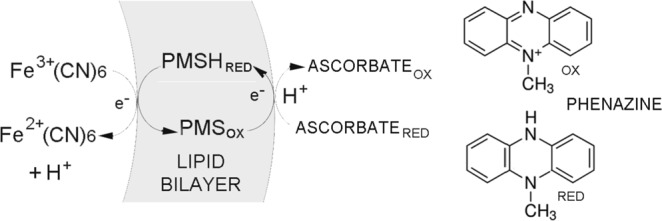
Establishing pH gradients in liposome preparations. Phenazine addition as phenazine methosulfate (PMS) was used as a positive control for the system. The oxidized and reduced forms of phenazine are shown on the right. Note that upon reduction a hydrogen is added to the nitrogen atom on the central ring. The hydrogen is released as a proton when the phenazine is oxidized by the ferricyanide in the liposomes, resulting in a decreased pH.

The ability of phenazine methosulfate (PMS) to act as an electron carrier and generate a pH gradient is shown in Fig. 2. When PMS is reduced by ascorbate into PMSH, the PMSH diffuses to the inner surface of the lipid bilayer where it donates electrons to ferricyanide contained by the liposomes, releasing protons and lowering the internal pH.

**Figure 3:**
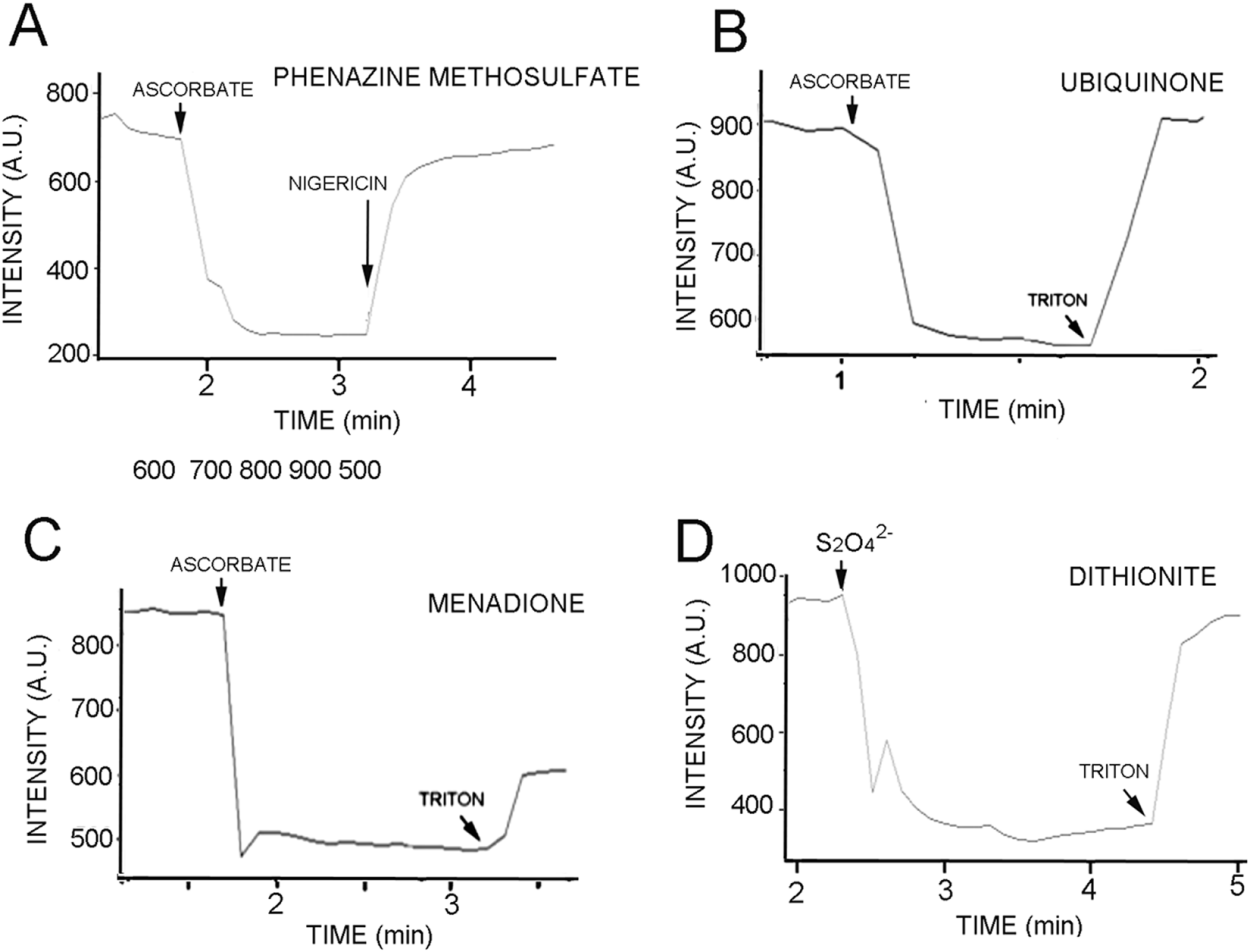
(**A**) Phenazine methosulfate was tested for its ability to produce a proton gradient across a liposome bilayer. The addition of ascorbate had no effect on fluorescence unless phenazine methosulfate was added to the mixture. The addition of ascorbate then caused fluorescence to become quenched by 72%. Quenching was reversed with the addition of nigericin, an antibiotic that allows proton gradients to equilibrate with potassium ions in the medium. As controls, ubiquinone and menadione (**B**,**C**) were used to test whether insoluble quinones could become embedded in lipid membranes during liposome preparation and function as electron carriers. (**D**) Demonstrates that sodium dithionite, a reduced sulfur compound, could also serve as a source of electrons in the system. In this case, PMS had already been added to the system to serve as an electron carrier.

The initial solution was 1.0 mL of ferricyanide-containing liposomes buffered at pH 7.4, 5 μM 9-aminoacridine and 4 μM phenazine methosulfate. The addition of 2 mM ascorbate caused fluorescence quenching to decrease approximately 72% (Fig. 3A). Nigericin is an antibiotic that discharges pH gradients by exchanging protons for potassium ions across the lipid bilayer^13^. We therefore used nigericin to confirm that pH gradients were in fact generated by the system. When nigericin (50 μg) was added, the fluorescence intensity increased to the original level, reflecting the decay of the pH gradient. For convenience, Triton X-100 rather than nigericin was used to discharge pH gradients in later experiments. The amounts added were just sufficient to increase permeability without disrupting the liposome membranes.

PMS is not a quinone, so two known quinones were used as standards to test whether they could act as carriers and develop pH gradients across the liposome membranes. Ubiquinone is a common electron carrier that is active in many eukaryotic mitochondrial electron transport chains where it accepts and transports electrons and protons between protein complexes to pass protons from the mitochondrial matrix to the intermembrane space^14^. Ubiquinone was embedded in liposome membranes in a 1:100 ubiquinone to lipid mass ratio as described in the Methods section. Its ability to generate a pH gradient across a membrane was tested by adding ascorbate to the external solution containing the liposomes. Figure 3B shows a typical result in which addition of 4 mM ascorbate caused a decrease in fluorescence intensity from 900 AU to 540 AU, representing a 40% quenching of fluorescence. The non-ionic detergent Triton X-100 is commonly used to make membranes permeable to ionic flux by partitioning into the hydrocarbon chains and producing hydrated defects through which ions can pass. When 1% Triton X-100 (50 μL) was added to the mixture, quenching was completely reversed.

A second quinone was also tested as an electron carrier in the model system. Menadione (2-methyl-1,4-naphthoquinone) was added to the lipids prior to liposome preparation in a 1:100 ratio of menadione to lipid by mass. When ascorbate was added to the liposomes in the model system containing menadione, 9AA fluorescence was quenched by 45% (Fig. 3C). Addition of Triton X-100 partially reversed the quenching.

### Sodium dithionite: an alternative reducing agent

Ascorbic acid is not a plausible prebiotic source of reducing power, but reduced forms of sulfur such as hydrogen sulfide, sulfite, thiosulfate and dithionite can serve in this regard. Therefore sodium dithionite (S_2_O_4_)^2−^ was tested as an electron donor to model the kinds of reducing agents that may have been present in prebiotic geochemical environments. In the liposome system containing ferricyanide as an oxidant and 4 μM PMS as an electron carrier, the addition of 4 mM dithionite resulted in fluorescence quenching of 72% which was reversed by adding 50 μL of 1% Triton X-100 (Fig. 3D).

### Mighei, Murchison, and Murray meteorite extracts

The discovery of different PAH and quinone compounds in carbonaceous meteorite samples suggests that there may be compounds capable of acting as electron carriers across a membrane if an electron acceptor and electron donor are present on opposite sides of the membrane. If these compounds were able to produce an electrochemical proton gradient across the bilayer, the potential energy of the gradient could be used to drive a variety of energy-dependent reactions.

The organic-soluble compounds of Murchison, Murray and Mighei meteorite samples were extracted in chloroform and tested for electron carrier activity. Because the compounds are relatively insoluble in water, they were added to the chloroform solution of lipids before liposome preparation to ensure that they were embedded in the hydrophobic lipid bilayer of the liposomes. This allowed any lipid-soluble compounds in the meteorite samples to insert into the lipid bilayer in the same manner as ubiquinone and menadione. Ascorbate could then be added to the liposome preparation to initiate the electron transport reaction.

**Figure 4:**
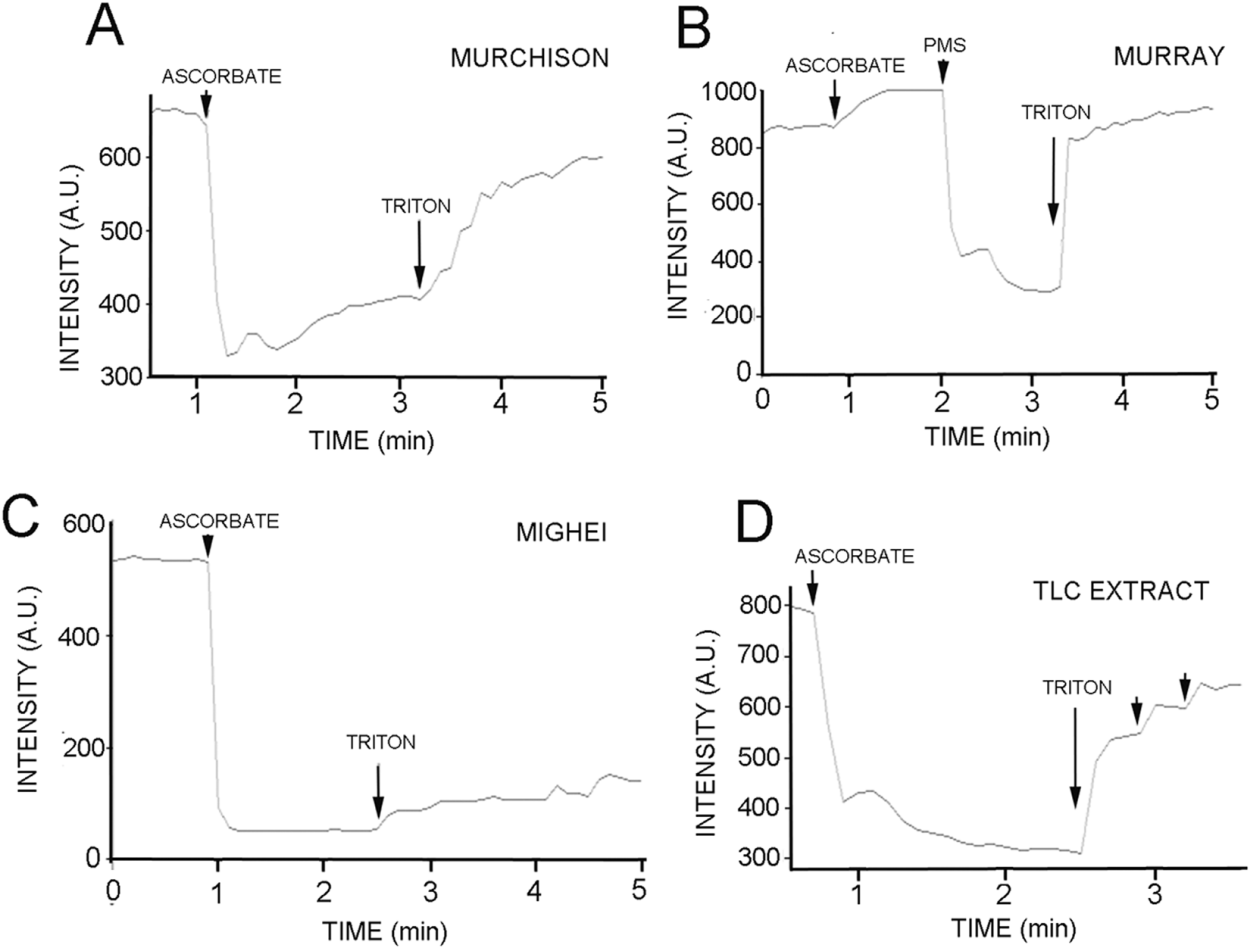
(**A**) Evidence for electron carriers in meteorite extracts. The nonpolar compounds of the Murchison meteorite organic extract were allowed to embed in liposome membranes before testing for electron carrier activity. The addition of ascorbate caused a fluorescence quenching of 56% which could be reversed with Triton X-100. The organic compounds of the Murray meteorite sample did not produce quenching when ascorbate was added (**B**) but addition of PMS led a quenching of fluorescence by 75% which could be reversed with Triton X-100. When the organic extract of the Mighei meteorite was added to the model system, an 86% quenching of fluorescence resulted (**C**) but could not be reversed with the addition of Triton X-100. The Murchison meteorite organic extract was partially purified by thin layer chromatography and one portion (see Fig. 5) also produced fluorescence quenching of 63% that was reversed by adding Triton X-100 (**D**).

Figure 4A shows the result of adding ascorbate to the ferricyanide-containing liposomes prepared with Murchison extract added. Fluorescence decreased from 720 AU to 320 AU, a quenching of 56%, and addition of 1% Triton X-100 reversed the quenching. It is interesting that the fluorescence intensity slowly began to increase even before Triton addition. This is probably due to other compounds in the meteorite extract that increased the permeability of the lipid membranes to protons.

We observed that the organic extract of the Murray meteorite apparently lacked compounds capable of acting as electron carriers (Fig. 4B). When 2 mM ascorbate was added, fluorescence was not quenched, but instead increased slightly. As a control, 4 μM PMS was added to the cuvette after the addition of ascorbate, resulting in fluorescence quenching of 75%, and addition of Triton X-100 again reversed the quenching. The Murray result served as a useful negative control, confirming that the quenching was not simply due to the addition of ascorbate.

When the organic compounds of the Mighei meteorite sample were used, addition of ascorbate caused the fluorescence intensity to decrease from 570 AU to 80 AU, representing a significant quenching of 86% (Fig. 4C). However, addition of 1% Triton X-100 in volumes from 10 μL up to 100 μL only slightly reversed the quenching.

### Purifying potential carriers

**Figure 5:**
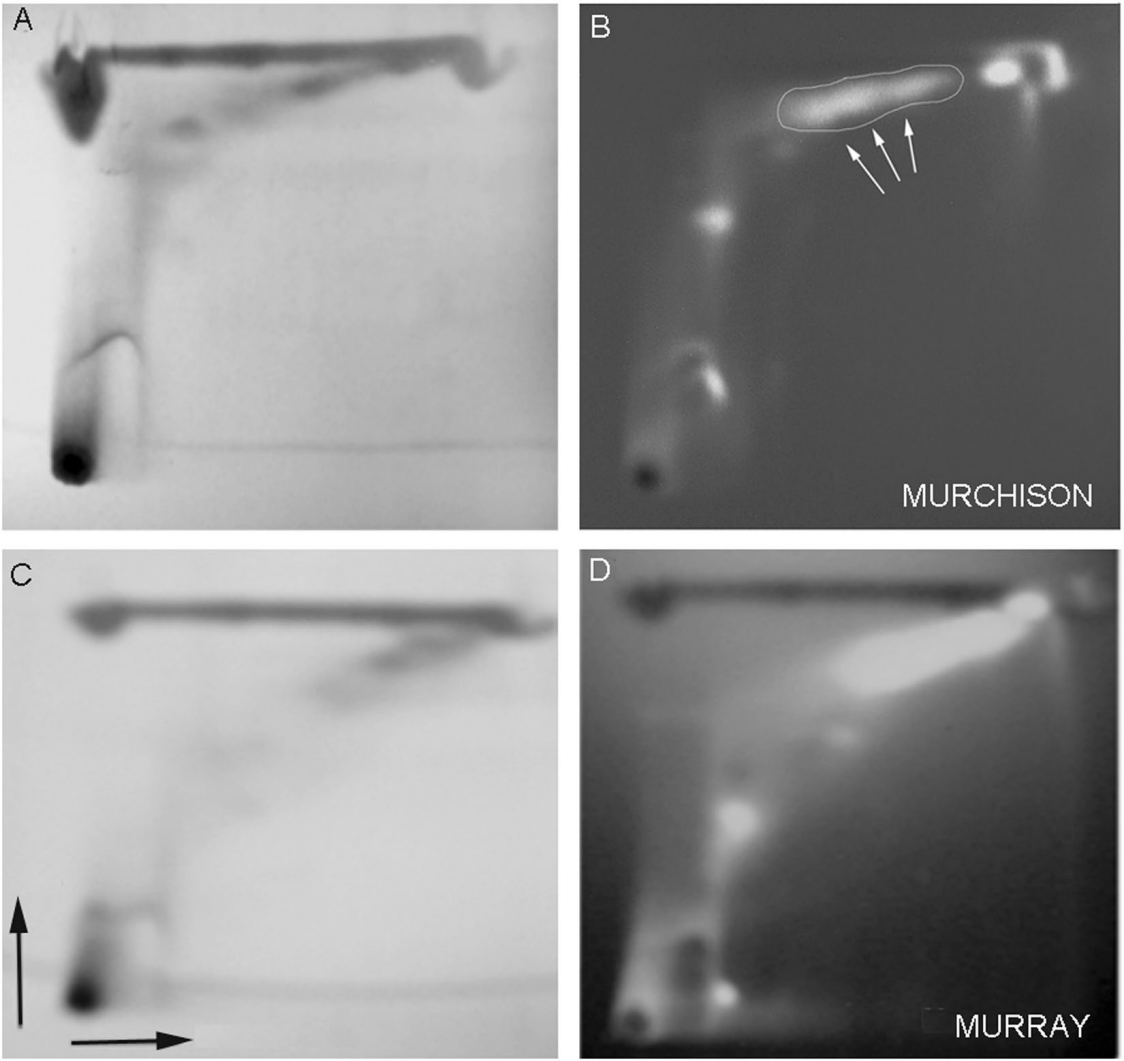
Purifying meteorite extracts by thin layer chromatography. Approximately 0.5–1 mg of Murray and Murchison meteorite samples dissolved in chloroform was spotted at the origin of silica gel TLC plates, then developed with 4:1 hexane:diethyl ether and chloroform in the x and y dimensions respectively (black arrows). (**A**) Shows the Murchison plate exposed to short wave UV light (254 nm), and (**B**) shows the same plate exposed to long wave UV light that excited fluorescence (~360 nm). The circled area (white arrows) was scraped and eluted for analysis by mass spectrometry. (**C**) Shows the Murray plate exposed to short wave UV light, and (**D**) shows the same plate exposed to long wave UV light.

Because the organic extracts of the Murchison and Murray meteorite samples contain numerous other compounds, including elemental sulfur, samples were separated by thin layer chromatography as described in Methods. When illuminated with short-wave UV light (254 nm) dark spots representing absorption were observed at the origin, in the middle of the TLC plate, and along the top of the plate (Fig. 5A,C). Under long-wave UV light (360 nm), fluorescent streaks were observed in some, but not all of the same regions (Fig. 5B,D).

The center of the Murchison plate revealed an area of interest in which fluorescent quinones and other semi-polar compounds were expected to migrate. To test for electron transport activity, the area indicated by arrows was scraped off as a powder, dissolved in chloroform and mixed with the phospholipid used to prepare liposomes. As shown in Fig. 4D, the addition of ascorbate caused 9AA fluorescence to be quenched by 63%, which was reversed by addition of Triton X-100.

Table 1 summarizes the magnitude of pH gradients calculated from 9-aminoacridine quenching and estimated volume ratios for all of the experiments. We conclude that the Murchison contains compounds, presumably quinones, that can function as electron carriers and develop pH gradients. The compounds can be partially purified by thin layer chromatography. Such compounds are apparently absent in the Murray meteorite. The Mighei meteorite extract produced a dramatic quenching of fluorescence upon addition of ascorbate (Fig. 4C) but the quenching could not be released by Triton addition so it might not be related to the development of a pH gradient.

**Table 1:** The calculated ΔpH magnitudes developed by transmembrane electron transfer across liposome membranes from external ascorbate to internal ferricyanide.

Sample	Lipid concentration	% Quenched	ΔpH
PMS	0.11 mg/mL	72%	2.71
PMS, Dithionite	0.11 mg/mL	72%	2.71
Ubiquinone	0.11 mg/mL	40%	1.88
Menadione	0.11 mg/mL	45%	1.93
Murchison	0.33 mg/mL	56%	1.91
Murray	0.11 mg/mL	0%	0
TLC sample, Murchison	0.33 mg/mL	63%	2.03
No electron carrier	0.1 mg/mL	0%	0

In one instance, dithionite replaced ascorbate.

### Gas chromatography-mass spectrometry (GC-MS)

In a preliminary experiment, the area of interest circled in Fig. 5B was scraped off the 2-D TLC plate, dispersed in acetonitrile and centrifuged for 5 minutes at 10,000 rpm to remove the silicic acid powder. An aliquot of the supernatant was injected into a Thermo Fisher LTQ-MS mass spectrometer to determine molecular weight of isolated compounds. This instrument detects molecular ions, and one of the peaks had an m/z of 171.9 which corresponded to 2-methyl naphthoquinone that we had already established as a possible electron carrier (Fig. 4D). Therefore we initiated a more extensive examination of meteoritic compounds in which mass spectrometry with an Agilent 5975 GC-MS instrument was used to confirm whether quinones were present in the Murchison and ALH 85013 extracts.

**Figure 6:**
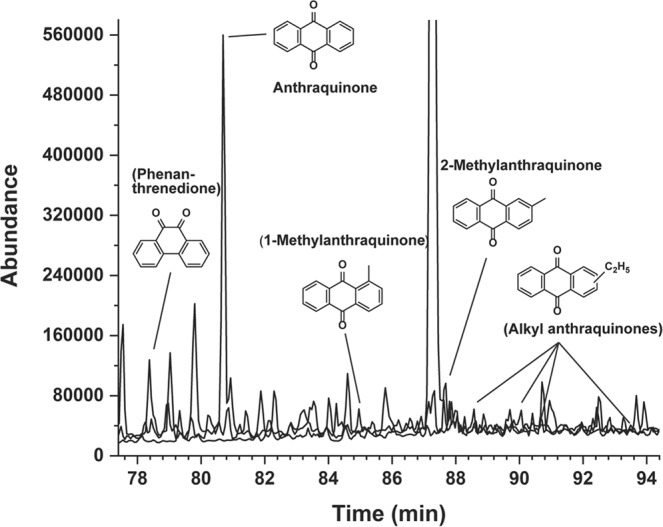
Quinones in the Murchison meteorite. Three individual traces are superimposed that represent single ion searches for the masses (molecular ions) of indicated quinones: m/z 208, anthraquinone (9,10-anthracenedione) and phenanthrenequinone (9,10-phenanthrenedione); m/z 222 (2-methylanthraquinone and isomer); m/z 236 (dimethyl anthraquinone isomers). Compounds in parentheses are tentative: the remaining compounds are definite identifications having GC retention times (x-axis) that match those of authentic standards. The mass spectra of phenanthrenequinone and 1-methylanthraquinone are exact matches of the mass spectra of the corresponding compound in the NIST (National Institute of Standards and Technology) mass spectral database, and the mass spectra of the alkyl anthraquinones are similar to those analyzed laboratory standards of dimethyl or ethyl (i.e., C_2_H_5_) anthraquinone derivatives.

**Figure 7:**
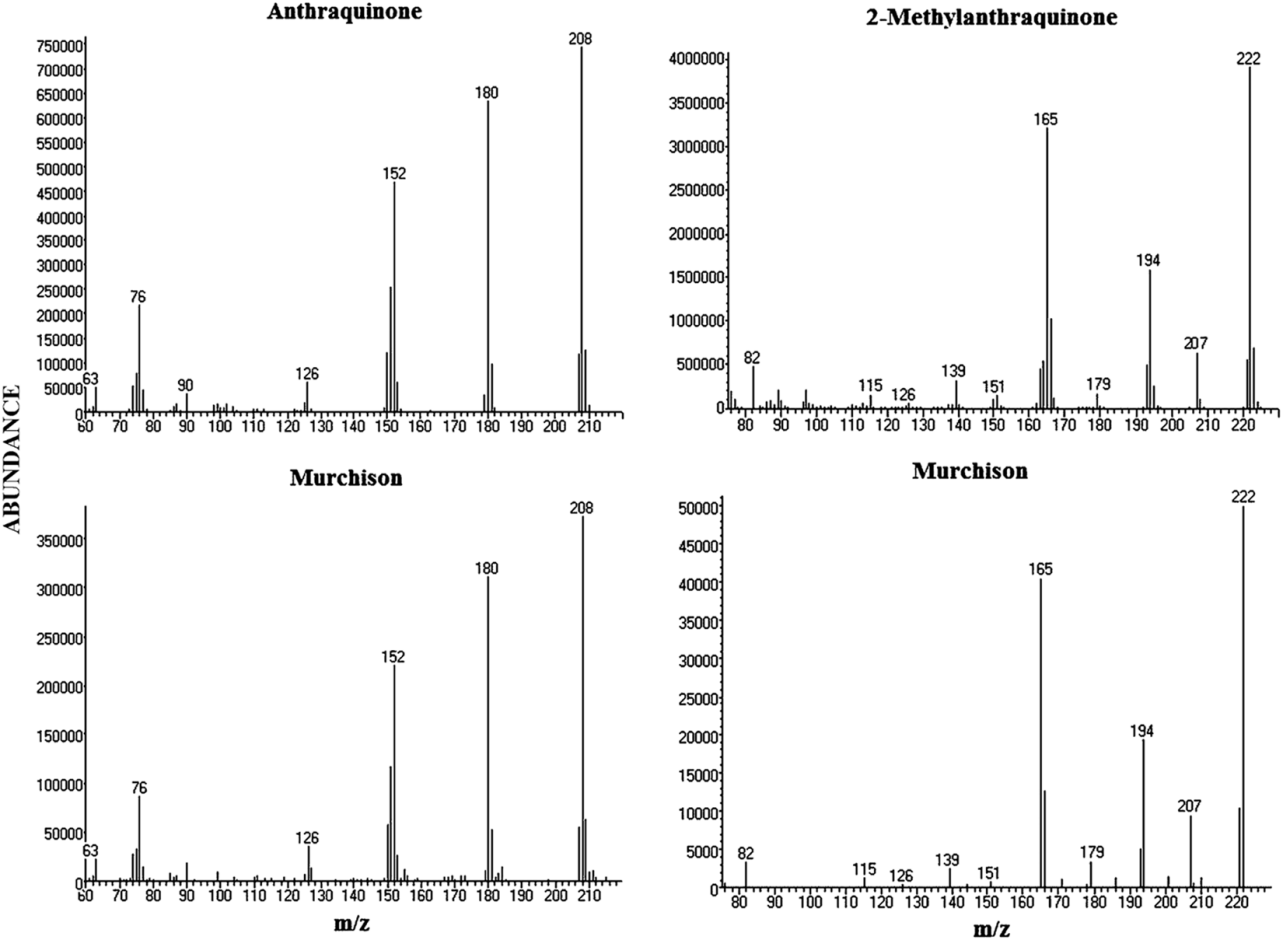
Mass spectra of anthraquinone and 2-methylanthraquinone and corresponding compounds from the Murchison meteorite. The two upper spectra show standards, and the lower spectra were from specific peaks in the GC chromatogram. The mass spectrum of Murchison’s 2-methylanthraquinone was obtained in selected ion monitoring (SIM) mode. In SIM, only a limited number of masses (mass-to-charge) are detected, which increases sensitivity.

**Figure 8:**
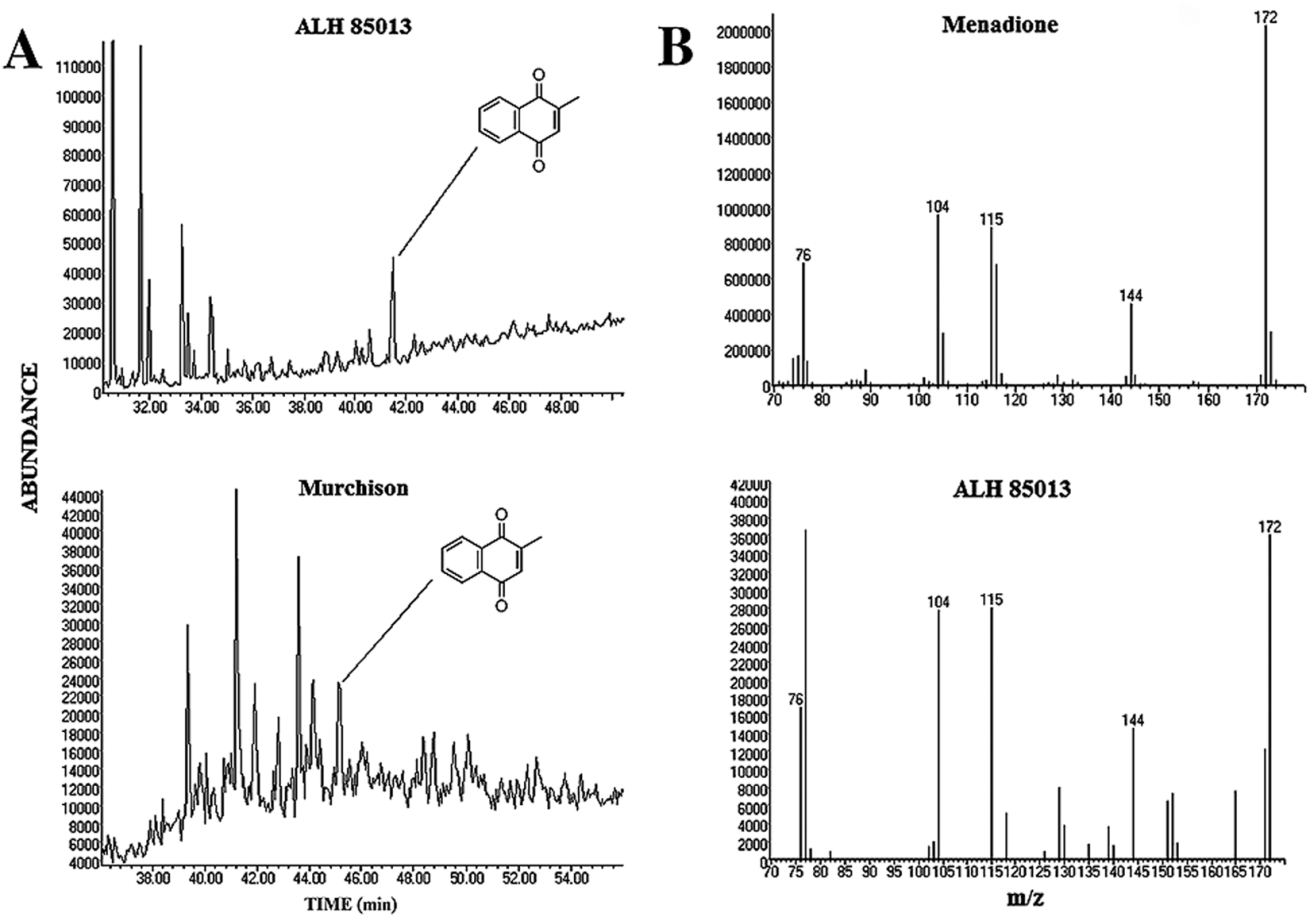
(**A**) Menadione (2-methylnaphthoquinone) in the ALH 85013 and Murchison meteorites. Traces represent single ion searches for the molecular ion (m/z 172) of 2-methylnaphthoquinone. (**B**) Mass spectra of menadione (2-methylnaphthoquinone) and corresponding compounds from ALH 85013. The mass spectrum of ALH 85013 was obtained in SIM mode. Additional fragments are due to co-elution of other meteoritic compounds. The Murchison and ALH 85013 GC temperature programs were different, hence the difference in the retention times of menadione (see Methods section).

Figures 6, 7, 8 and Table 2 show results of the GC-MS analyses of individual quinones from the Murchison extract. Because the extracts from the Murray and Mighei meteorites did not appear to have compounds capable of acting as electron carriers, we extended our study to extracted organics from the Antarctic carbonaceous meteorite ALH 85013. Multiple quinones in the Murchison extract were revealed by gas chromatography (Fig. 6), but only two were identified in ALH 85013, perhaps because a smaller sample was analyzed. The most abundant quinones in Murchison were anthraquinone (9,10-anthracenedione) and methylanthraquinone (2-methyl-9,10-anthracenedione) in the nmole/gram and pmole/gram range, respectively. Anthraquinone and 9,10-phenanthrenedione were also identified previously in Murchison^3^. The mass spectra shown in Fig. 7 confirm the presence of anthraquinone and 2-methyl anthraquinone in the Murchison meteorite, and Table 2 summarizes quantitative results.

**Table 2:** Quinones identified in the Murchison meteorite.

Compound	Murchison (nmoles/gram)
2-Methyl-1,4-naphthoquinone	0.3
9,10-Anthraquinone*	2.0
(9,10-Phenanthrenedione)	nd
2-Methyl-9,10-anthraquinone*	0.5
(1-Methyl-9,10-anthraquinone)	nd
(C_2_H_5_-Anthraquinone Isomers)	nd

Also present in the ALH 85013 carbonaceous meteorite. nd = Present but abundance not determined. in some cases, the corresponding standard was not available at the time of analysis. Standards for 2,3-dimethylanthraquinone and 2-ethylanthraquinone were run but low abundances of the corresponding meteoritic compounds led to lower quality mass spectra and quantitative analysis.

The gas chromatogram in Fig. 8 shows a distinct peak for 2-methylnaphthoquinone (menadione) in both the Murchison and ALH 85013 extracts which was confirmed by the mass spectra associated with these peaks.

## Discussion

Given a redox gradient across membranes, quinones associated with electron transport systems are coupled to proton transport such that a chemiosmotic proton gradient develops in microbial, mitochondrial and chloroplast membranes. The energy available in the gradient is coupled to an ATP synthase that produces most of the adenosine triphosphate used as an intracellular energy currency. Ubiquinone and plastoquinone incorporated in the membranes of mitochondria and chloroplasts serve as electron carriers that generate proton gradients, but these are synthesized enzymatically and would not have been available to the earliest forms of microbial life. The aim of our research was to determine whether simpler quinones could have been present in the prebiotic environment to serve as electron carriers. The liposome model system described here allowed us to test standard quinones for this capacity and then compare the results to meteorite extracts under the same conditions. We note that in order to generate a proton gradient, the membranes cannot be “leaky” to protons. Therefore the liposome were composed of phospholipids that were sufficiently impermeable to protons to maintain a pH gradient.

A phylogenetic analysis of quinones present in electron transport chains of prokaryotes led to the proposal that menadione (2-methyl naphthoquinone) may have been one of the first quinones involved in electron transport chains in an anaerobic environment before the Great Oxygenation Event 2.3 billion years ago^2^. We therefore tested whether menadione could act as an electron carrier in the liposome model system and found that a pH gradient of 1.9 pH units was generated which could be partially reversed by addition of Triton X-100 to make the membranes permeable to hydrogen ions (Fig. 3C). A pH gradient of similar magnitude was also produced by ubiquinone (Fig. 3B).

Although we used ascorbic acid as an electron donor in the model system, it is unlikely to have been present in the suite of compounds available to participate in prebiotic redox reactions. Reduced sulfur compounds are potential reducing agents, but they must be relatively impermeable to lipid bilayers if a redox gradient is to be established. This requirement excludes hydrogen sulfide (H_2_S) a small neutral molecule which, like water molecules (H_2_O), is expected to readily diffuse across membranes. On the other hand, sulfur dioxide is abundant in volcanic emissions, forming sulfurous acid and sulfite when dissolved in water. Sulfite anions (SO_3_)^−^ have reducing power and are also relatively impermeable. We therefore tested a similar sulfur compound called dithionite (S_2_O_4_−)^2−^ as an electron donor in our system. As shown in Fig. 3D, when sodium dithionite was added to liposomes with phenazine methosulfate as a carrier we observed that a gradient of 2.7 pH units rapidly developed, similar to that observed with ascorbate as an electron donor. We conclude that reduced sulfur compounds in the prebiotic environment could serve as effective reducing agents for production of pH gradients, and that ascorbic acid can be a convenient alternative donor for the experimental system.

The results with ubiquinone and menadione confirmed that the liposome system could be used to explore whether meteoritic quinones could also function as carriers to couple electron transport across lipid bilayer membranes and produce proton gradients. We decided to investigate samples of four meteorites that were available for the study. Murchison, Murray, Mighei and ALH 85013 all belong to the CM (“M” for Mighei) group of carbonaceous meteorites (“chondrites”). They are also petrologic type 2, which means that they experienced significant aqueous alteration on their parent asteroid bodies. After establishing that the model system could be used to monitor pH gradients, we extracted samples of the Murchison, Murray and Mighei meteorites with an organic solvent system. The Murchison and Murray extracts clearly contained compounds that could serve as electron carriers and generate pH gradients (Fig. 4A,B). The Mighei extract also quenched fluorescence, as though a pH gradient was present (Fig. 4C), but the quenching could not be reversed with Triton addition so we did not pursue this observation further.

In experiments with the Murchison extract (Fig. 4A), we noted that there was a slow reversal of quenching which suggested that the membranes were allowing the pH gradient to decay as hydrogen ions leaked out. This was likely to be a consequence of other compounds in the extract that made the liposome membranes more permeable to protons. To remove potential contaminants, we used 2-dimensional thin layer chromatography to separate the Murchison organics according to their polarity and observed a spot in the center-top region of the chromatogram which absorbed short-wave UV light but was also fluorescent when excited by long-wave UV light (Fig. 5B). We guessed that these properties are consistent with what would be expected for a semi-polar PAH derivative containing quinone functional groups. When the compounds in this region were embedded in liposome membranes and tested for electron transport activity using the model system, the fluorescence quenching was similar in magnitude to what was observed with the Murchison organic extract but the purified material did not cause the decay of quenching (Fig. 4D). Triton addition reversed the quenching, as expected.

The fact that two known quinones as well as compounds in meteoritic extracts could generate pH gradients led us to analyze extracts of the Murchison and an Antarctic CM2 meteorite by gas chromatography-mass spectrometry. The GC chromatogram clearly showed that small amounts of anthracenedione and 2-methyl anthracenedione were present in the Murchison (Fig. 6) but the smallest member of the homologous series (1,4-naphthoquinone) was not detected. Because 1,4-naphthoquinone sublimes at 100 °C (STP), this could simply be due to sublimation of naphthoquinone from interstellar dust particles in the solar nebula that accreted to form the parent body of carbonaceous chondrites. This possibility is supported by the fact that the methylated member of the series, 2-methyl-1,4-naphthoquinone, is present at ~0.3 nmole/gram, but still significantly less than the larger compound anthraquinone (~1.6 nmole/g, Table 2). This is the reverse for most homologous series of compounds in meteorites that typically show a decrease in abundance with increasing carbon number^8^. An alternative is that naphthalene and anthracene quinones could have been synthesized by different chemical processes, either on interstellar dust or the parent bodies of the meteorites. For instance, oxidation of anthracene yields anthraquinone more readily than naphthalene yields naphthoquinone^15^. This mechanism of formation of certain quinones is plausible given the ubiquitous presence of PAHs in carbonaceous meteorites.

A recent study reported another mechanism by which transmembrane pH gradients can be generated^16^. In this, an encapsulated iron sulfur peptide mediated the transfer of electrons from a reduced compound such as NADH to hydrogen peroxide. The hydroxide anion (OH^−^) that was produced increased the internal pH. Although small pH gradients could be detected, the reaction was much slower (hours) than the quinone-mediated reaction described here (seconds).

We conclude that compounds capable of facilitating electron transfer across membranes were likely to have been delivered by meteoritic infall and available in the prebiotic environment. Furthermore, if membranous compartments happened to encapsulate a redox gradient such as external sulfite and internal ferric iron, the quinone compounds would mediate the development of proton gradients. To our knowledge, this is the first report that quinones present in carbonaceous meteorites can serve a functional role in electron transport. Although our results show a correlation between the presence of quinones in carbonaceous meteorites and their possible involvement in electron and proton transfer across membranes, we have not demonstrated a causal relationship. There is still much to learn before we can determine whether such systems would spontaneously emerge, but the results reported here add quinones to the list of essential ingredients.

## Methods

### Liposome preparation

Liposomes were prepared from a 10:1 mole ratio mixture of 1,2-dioleoyl-sn-glycero-3-phosphocholine (DOPC) and phosphatidylglycerol (PG). The PG added a negative charge to the liposome surfaces that prevented them from aggregating. The DOPC and PG were mixed in a chloroform solution, then the chloroform was evaporated to form a lipid film. A solution of 33 mM K_3_Fe(CN)_6_, (potassium ferricyanide) in 10 mM phosphate buffer (pH 7.4) was added to the film to produce a final lipid concentration of 1 mg/mL, followed by bath sonication for 30–60 seconds to disperse the lipid as small liposomes^11^. When the liposome fraction was examined with 400X phase microscopy, a majority of 0.2–1 μm unilamellar vesicles were observed mixed with a few larger multilamellar vesicles. Although the small vesicles could be seen by eye, micrographs could not be taken due to their brownian motion. All experiments using the liposomes were performed within 5 hours of preparation.

In order to remove the external ferricyanide, the sonicated suspension (1 mL) was put through a 1 × 10 cm size-exclusion chromatography column of Sephadex G-50, which was equilibrated with pH 7.4 buffer consisting of 50 mM K_2_SO_4_ in 10 mM phosphate buffer to balance the osmotic concentration of ionic species across the liposome bilayer. The turbid liposome fraction was collected in 3–5 mL of the eluted volume.

The proton gradient that developed with phenazine methosulfate as an electron carrier was used as a positive control. If the neutral form of monoamines can diffuse across lipid bilayer membranes of liposomes, then the monoamines distribute between the internal and external volume in response to pH gradients when an acidic interior is present^11^. In the acidic interior of the liposome, the protonated form of the amine is positively charged and cannot cross the membrane, so it accumulates inside and its fluorescence is quenched. The magnitude of quenching is related to the distribution of the amine between the internal and external volume.

Fluorescence was measured with the kinetics module of an Agilent Cary Eclipse Fluorescence Spectrophotometer. Emission and excitation wavelengths of 9-aminoacridine (9AA) were determined to be 455 nm for emission and 326 nm for excitation. The wavelengths were established by scanning with excitation and emissions slits set at 5 nm.

To test the effect of the addition of an experimental electron carrier, 9AA was added to a final concentration of 4 μM in 1 mL of the ferricyanide containing DOPC/PG liposomes in a quartz cuvette. When PMS was used as the electron carrier, the fluorescence of the 9AA in the cuvette was monitored and recorded over 5 minutes, during which 4 mM ascorbate, 4 μM PMS were added with 1–2 minute intervals in between to allow the reactions to proceed. The addition of ascorbate caused a pH gradient to be produced as shown in Fig. 3, with the internal volume becoming acidic while the external phosphate buffer remained at pH 7.4. Addition of nigericin was used initially to confirm that pH gradients were being generated. This antibiotic specifically allows hydrogen ions to exchange for potassium ions in the medium. After this had been established, addition of Triton X-100 was employed as a convenient way to discharge pH gradients. We were careful to use a concentration of Triton that permeabilized the membranes but did not cause them to be dispersed into mixed micelles.

### Estimating [A_i_/A_o_], V_o_/V_i_, and ΔpH

Monoamines such as 9-aminoacridine are fluorescent, but the fluorescence is quenched at high concentrations which allows the ratio of [A_i_]/[A_o_] to be determined. The distribution of 9-aminoacridine across a membrane in relation to an existing pH gradient can be expressed in logarithmic form as ΔpH = log [A_i_]/[A_o_] + log Vo/Vi where A_i_ and A_o_ are the total amounts of 9-aminoacridine in the respective internal and external volumes of liposomes, and V_o_/V_i_ is the ratio of the external volume to the internal liposome volume. The amount of quenched fluorescence is directly related to the amount of 9-aminoacridine that had accumulated in the internal volumes of liposomes after a pH gradient was produced. As a result, [A_i_/A_o_] is equivalent to Q/(100 − Q), where Q is the percent quenching recorded.

V_o_/V_i_ was determined in two ways. First, it is possible to calculate the encapsulated volume of a 1 mg/mL lipid concentration from the mean diameter of sonicated liposomes and an area per phospholipid molecule in a lipid bilayer (0.55 nm^2^). This yields a V_i_ of 64.2 μL and a total V_o_/V_i_ of 15.6. Using this calculated value with the [A_i_]/[A_o_] from the different electron carrier experiments, ***Δ***pH values ranging from 2–3 pH units were estimated. For instance, if the total internal volume of liposomes is one microliter in one milliliter of a buffer solution, pH 8, and half the fluorescence is quenched, [A_i_]/[A_o_] = 1, and the ratio of the outer to internal volume V_o_/V_i_ = 1000. The ΔpH is then log [A_i_]/[A_o_] + log V_o_/V_i_ = 3 pH units.

A second estimate of V_o_/V_i_ was obtained in a separate experiment by measuring the volume of trapped internal contents using pyranine, a fluorescent probe. This experiment showed that ~5.2% pyranine was captured by the liposomes at a concentration of 1 mg/mL, which is equivalent to a V_o_/V_i_ of 19.2. Because V_o_/V_i_ is expressed as the log base 10, the discrepancy between the calculated and measured volume ratio only affects the estimate of ΔpH by a tenth of a pH unit. Lipid concentrations in a range of 0.06–0.24 mg/mL should be used to measure pH gradient magnitudes that exist within a range of 2–3 pH units. The 1 mg/mL original lipid concentration works because the liposomes are diluted during filtering with Sephadex G-50 size-exclusion chromatography to remove external ferricyanide. The diluted preparations have lipid concentrations within the appropriate range. For example, a 1:10 dilution of a 1 mg/mL lipid mixture yields a 0.1 mg/mL lipid concentration with a V_o_/V_i_ of 19.2, and this is sufficient for maximal quenching of 9AA fluorescence as it accumulates in the liposomes^17^.

### Meteorite extraction for thin layer chromatography analysis

Samples of the Murchison, Mighei, and Murray meteorites were kind gifts of the Field Museum in Chicago. They were provided in 1989 for a previous study and were stored in aluminum foil in a closed glass container since that time. A mortar and pestle were cleaned with deionized water, ethanol, and 2:1 chloroform:methanol before use to ensure that no contaminants were present in the extraction process. The Murchison, Mighei, and Murray meteorite samples (1–2 grams) were ground to a powder in a mixture of 0.1 M HCl (1 mL) and 2:1 chloroform:methanol (3 mL). After centrifugation in glass centrifuge tubes to separate the chloroform and aqueous phases, the aqueous phase was removed by pipeting and the chloroform phase could be decanted from the mineral pellet and stored in glass vials.

### Thin layer chromatography

To fractionate the meteorite extracts in chloroform, a total of 1 mL of each extract was applied to a 10 cm × 10 cm silicic acid TLC plate containing a mineral fluorophore that fluoresced when illuminated with 254 nm UV light. The volume was added in increments (10 μL) and was allowed to evaporate between each addition. For two dimensional TLC, the plate was developed in 4:1 hexane: diethyl ether in the first dimension, then turned 90 degrees and developed in chloroform in the second dimension. This process separated organic components according to their polarity. The TLC plates were illuminated by two wavelengths of ultraviolet light in order to visualize the separated compounds. Short wave UV light at 254 nm caused the fluorophore in the plate to fluoresce, and organic compounds absorbing that wavelength showed up as dark spots or streaks. Elemental sulfur appeared as a long streak along the top of the plate. Nonpolar compounds like pyrene and fluoranthene migrated to the top-right side of the plate^9^ and were visualized by fluorescence activated by longer wavelength UV light in the 350 nm range. More polar fluorescent compounds migrated to the upper center of the plate, and we assumed that these included quinones because of their conjugated cyclic dione structure. This fluorescent region was marked, scraped off the aluminum backing, then extracted with 500 μL chloroform and concentrated to a 100 μL fraction for further analysis or addition to lipid solutions prior to preparation of liposomes.

### Meteorite extractions for GC-MS analyses of individual quinones

The Murchison meteorite sample was provided by the Center for Meteorite Studies - Arizona State University. ALH 85013 was provided by the Meteorite Working Group of NASA’s Johnson Space Center. Both meteorites are class CM2 chondrites, and ALH 85013 was extracted specifically for potential quinone components.

Finely ground powders of the Murchison and ALH 85013 were subjected to periods (20–30 min) of sonication-extraction in excess distilled dichloromethane. ALH 85013 was extracted twice (1 ml each), and Murchison five times (3 ml each). After each period of extraction the liquid was removed following centrifugation, and all extracts were combined. In the case of Murchison, to remove a portion of meteoritic elemental sulfur, ~267 μmoles (17 mg) of copper powder (Alfa Aesar, 100 mesh) were added to the combined extracts and the solution was allowed to sit for several hours before centrifugation. The liquid extracts of each meteorite were concentrated by evaporation at room temperature before injection. Aliquots of the extracts equivalent of 56 mg and 120 mg of ALH 85013 and Murchison powders, respectively, were injected. Identical treatment of a control, dichloromethane with and without copper, showed no contaminants.

### Gas chromatography-mass spectrometry (GC-MS) of individual quinones

Analyses were performed with an Agilent 6890 N GC (splitless mode) interfaced to an Agilent 5975B Mass Selective Detector (quadrupole mass spectrometer-EI mode). The GC was equipped with an Agilent J&W DB-17MS column (length: 60 m; diameter: 0.25 mm; film thickness: 0.25 μm). Typical GC parameters: injector temperature 300 °C with helium (UHP grade) as the gas carrier with a flow of 1.0 mL/min, a transfer line temperature of 300 °C. Typical GC temperature programs, for example in Figs 6 and 8A (Murchison): initial T, 35 °C – hold 0.5 min, then 4 °C/min to 175 °C-hold 20 min, 2 °C/min to 300 °C – hold 60 min. The GC program for Fig. 8B (ALH 85013): initial T, 35 °C – hold 0.5 min, then 4 °C/min to 300 °C-hold 60 min.

The mass spectrometer was set at an electron energy of 70 eV; quadrupole temp., 150 °C; source temp. 230 °C; carrier gas, helium; and operated in both full scan and selected ion monitoring (SIM) mode.

## Data Availability

Original results and data can be made available to interested parties.
